# The Structural and Functional Basis for Recurring Sulfa Drug Resistance Mutations in *Staphylococcus aureus* Dihydropteroate Synthase

**DOI:** 10.3389/fmicb.2018.01369

**Published:** 2018-07-17

**Authors:** Elizabeth C. Griffith, Miranda J. Wallace, Yinan Wu, Gyanendra Kumar, Stefan Gajewski, Pamela Jackson, Gregory A. Phelps, Zhong Zheng, Charles O. Rock, Richard E. Lee, Stephen W. White

**Affiliations:** ^1^Department of Chemical Biology & Therapeutics, St. Jude Children's Research Hospital, Memphis, TN, United States; ^2^Department of Microbiology, Immunology, and Biochemistry, University of Tennessee Health Science Center, Memphis, TN, United States; ^3^Structural Biology, St. Jude Children's Research Hospital, Memphis, TN, United States; ^4^Infectious Diseases, St. Jude Children's Research Hospital, Memphis, TN, United States; ^5^Pharmaceutical Sciences, University of Tennessee Health Science Center, Memphis, TN, United States

**Keywords:** infectious disease, antibiotics, drug discovery, fitness cost, kinetics, drug susceptibility, bacterial genetics

## Abstract

Staphylococcal species are a leading cause of bacterial drug-resistant infections and associated mortality. One strategy to combat bacterial drug resistance is to revisit compromised targets, and to circumvent resistance mechanisms using structure-assisted drug discovery. The folate pathway is an ideal candidate for this approach. Antifolates target an essential metabolic pathway, and the necessary detailed structural information is now available for most enzymes in this pathway. Dihydropteroate synthase (DHPS) is the target of the sulfonamide class of drugs, and its well characterized mechanism facilitates detailed analyses of how drug resistance has evolved. Here, we surveyed clinical genetic sequencing data in *S. aureus* to distinguish natural amino acid variations in DHPS from those that are associated with sulfonamide resistance. Five mutations were identified, F17L, S18L, T51M, E208K, and KE257_dup. Their contribution to resistance and their cost to the catalytic properties of DHPS were evaluated using a combination of biochemical, biophysical and microbiological susceptibility studies. These studies show that F17L, S18L, and T51M directly lead to sulfonamide resistance while unexpectedly increasing susceptibility to trimethoprim, which targets the downstream enzyme dihydrofolate reductase. The secondary mutations E208K and KE257_dup restore trimethoprim susceptibility closer to wild-type levels while further increasing sulfonamide resistance. Structural studies reveal that these mutations appear to selectively disfavor the binding of the sulfonamides by sterically blocking an outer ring moiety that is not present in the substrate. This emphasizes that new inhibitors must be designed that strictly stay within the substrate volume in the context of the transition state.

## Introduction

Sulfonamides are the oldest class of synthetic antibiotics. They have been in clinical use for over 75 years, with proven efficacy against many microbial infections (Domagk, 1935; Bermingham and Derrick, 2002). Sulfonamides target the enzyme dihydropteroate synthase (DHPS) that catalyzes a key step in microbial folate biosynthesis, the production of 7,8-dihydropteroate from *para*-aminobenzoic acid (*p*ABA) and dihydropterin pyrophosphate (DHPP). Sulfonamides exert their antimicrobial action in two ways, by directly competing with the substrate *p*ABA and through the formation of pterin-sulfa dead-end metabolic products (Roland et al., 1979). Prokaryotes and lower eukaryotes rely on this pathway for the *de novo* synthesis of folate that is a critically important cell metabolite, and disruption of folate biosynthesis therefore severely curtails their growth. In contrast, higher eukaryotes obtain folate directly from their diet and have dispensed with the pathway. The universal presence of DHPS in lower organisms and its absence in higher organisms explains why sulfonamides have been successful as broad-spectrum antimicrobials (Bermingham and Derrick, 2002).

Today, sulfonamides are mainly used in a fix dose combination with trimethoprim (TMP), a dihydrofolate reductase (DHFR) inhibitor. Co-trimoxazole, a combination of sulfamethoxazole (SMX), and TMP, is the most commonly prescribed. This cheap and orally bioavailable combination is used as a second-line therapy to treat a wide variety of bacterial infections including urinary tract infections (UTIs), bronchitis, traveler's diarrhea, and methicillin-resistant *Staphylococcus aureus* (MRSA) infections. Application of co-trimoxazole prophylaxis to prevent *Pneumocystis jirovecii* infections in immunosuppressed patients, such as those undergoing intensive cancer chemotherapy or with advanced HIV infections, has also emerged as a particularly important clinical application (Bermingham and Derrick, 2002).

The emergence of multidrug and pan resistant bacterial pathogens is an alarming and increasing phenomenon that requires immediate action (Boucher et al., 2009). To tackle this problem, we are revisiting previously identified antimicrobial targets and applying new strategies to develop inhibitors that are less prone to resistance mechanisms. Key to this approach is gaining an improved understanding of the targets' biochemical mechanisms, active site structures and resistance mechanisms. In many ways, DHPS is the perfect candidate for such an approach. Structurally and mechanistically, DHPS has been well characterized. The crystal structures of DHPS have been determined from 15 microbial species within the last 20 years, and more recent structural and computational studies from our group have revealed the ordered S_N_1 catalytic mechanism and the detailed configuration of the near transition state (Yun et al., 2012). These new insights have already enabled us to generate pyridazine derivatives with improved DHPS inhibition, identify allosteric inhibitors that hinder product release, and develop inhibitory pterin-sulfa conjugates (Zhao et al., 2012, 2016; Hammoudeh et al., 2014).

In this study, we focus on the structural and mechanistic basis of sulfonamide resistance in *S. aureus* DHPS (*Sa*DHPS), and analyze how recurring resistance mutations can selectively disfavor the binding of sulfonamides while retaining the necessary fitness of the enzyme. Crystal structures of DHPS containing pterin•PPi•Mg•*p*ABA and pterin•PPi•Mg•sulfonamide have revealed that *p*ABA and sulfonamides occupy the same locale in the near transition state (Yun et al., 2012). Our focus will be on this locale and how the resistance mutations modulate its structure and dynamics to selectively disfavor the binding of the drug. Our studies proceeded in three stages; (1) bioinformatics analysis to identify the key resistance mutations across organisms, (2) biochemical, resistance and fitness analyses of these mutations in DHPS from *S. aureus*, and (3) structural and computational analyses to understand the mechanistic basis of these mutations. Our goal is to use these results to support ongoing drug discovery efforts toward this enzyme and to develop lead compounds that are not cross-resistant to sulfonamides.

## Results

### Primary and secondary mutations confer sulfonamide resistance

The increasing prevalence of MRSA during the past two decades and the associated sequencing of clinical isolates has generated a large dataset of *Sa*DHPS sequence variations in the DHPS-encoding *folP* gene, including those that are found in sulfonamide resistant strains. We rigorously analyzed the available data up to and including 2014 to identify variations that are clearly associated with sulfonamide resistance. We identified two classes of resistance-associated mutations; primary mutations that are directly associated with sulfonamide resistance and secondary mutations that are only found in the presence of the primary mutations. An important goal of this analysis was to differentiate these mutations from the natural variations in *Sa*DHPS that are present in sulfonamide susceptible strains but do not directly contribute to resistance. The results of this survey are summarized in Table 1. F17L, S18L, and T51M emerge as primary mutations; F17L occurs at the highest frequency, and S18L and T51M were each observed in less than 1% of sequences surveyed as single variants. E208K and KE257_dup (a duplication/insertion of Lys-Glu at position 257) were categorized as secondary mutations. E208K is found with both F17L and T51M, and KE257_dup is only found with F17L. The primary mutation S18L is not found with either of the two secondary mutations. In an earlier study, Hampele and coauthors identified 15 mutations among nine sulfonamide-resistant MRSA clinical isolates that are not present in the sulfonamide susceptible *S. aureus* Rosenbach 25923 strain (Hampele et al., 1997). Although this study also identified F17L, T51M, E208K and KE257_dup, our analysis showed that the 11 remaining mutations are found in sulfonamide susceptible *S. aureus* strain NCTC 8325 and are apparently natural polymorphisms in *Sa*DHPS that do not contribute to sulfonamide resistance (Supplementary Table 1).

**Table 1:** Survey of DHPS variants and known resistance to sulfonamides.

**Sequence** **Background**	**25923**	8325^*^	**8325**	**25923**	**8325**	**8325**	**25923**	**8325**	**25923**	**8325**
Resistance Mutations	None	None	F17L KE257_dup	T51M E208K	T51M E208K	F17L E208K	F17L	F17L	T51M	S18L
% Sequences (*n* = 136)	28	49	3	8	1.5	3.7	3	2	0.7	0.7
Hampele Strain			Group 1	Group 2	Group 3	Group 4				
Hampele MIC (μg/mL)			256–>1024	256–>1024	>1024	>1024				
Sulfonamide resistant	No	No	Yes	Yes	Yes	Yes	ND	ND	ND	ND

Hampele strain group and MIC values have previously been published (Hampele et al., 1997).

* *Indicates that among the wild type folP genes surveyed and categorized as having an 8325 background, 31% lacked the natural variations T59S and L64M seen in the S. aureus strain Rosenbach 25923 (Supplementary Table 1)*.

**Figure 1:**
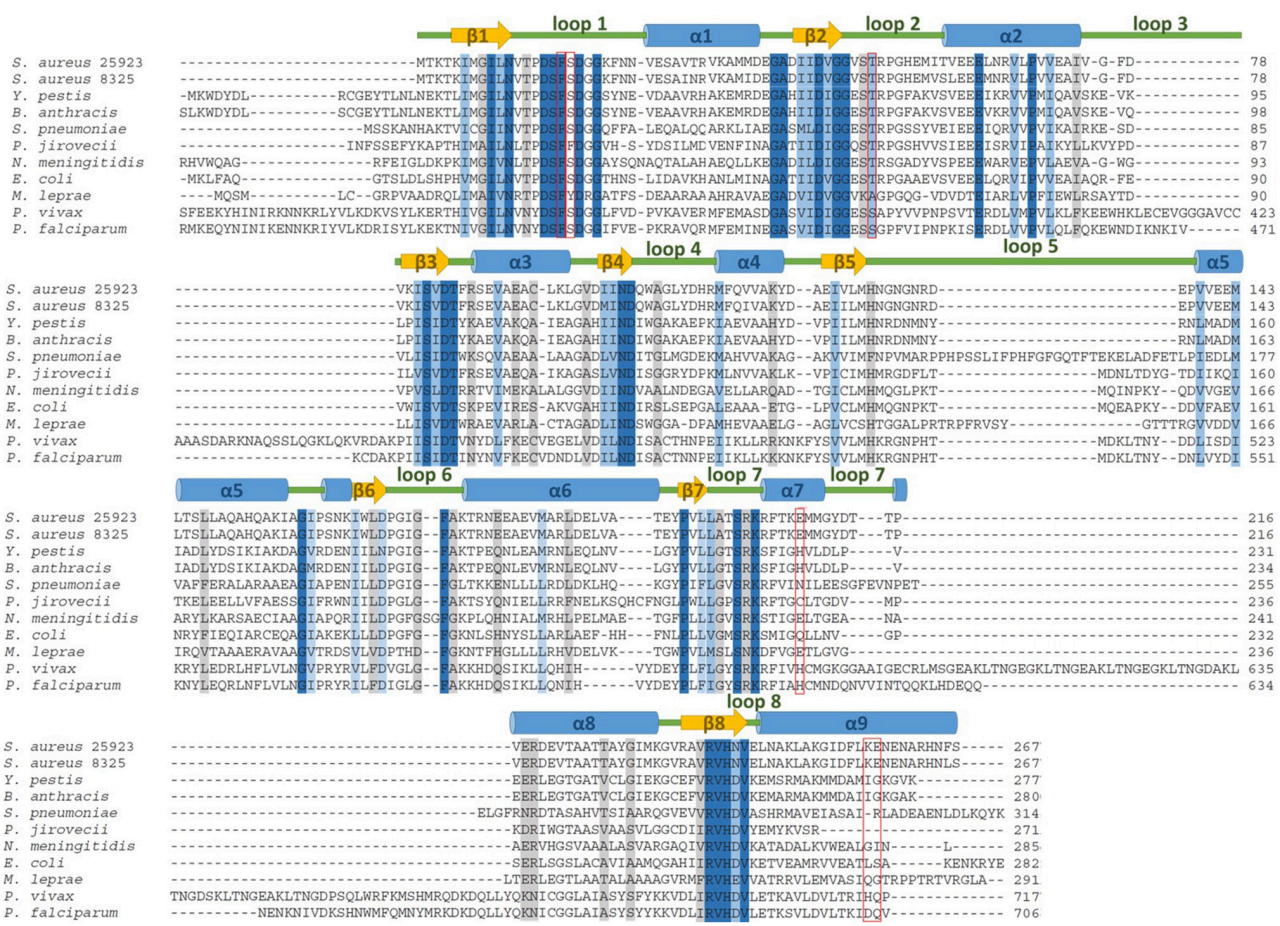
DHPS amino acid sequence alignment for *S. aureus* wild type representatives used in this study and nine other clinically relevant microbial pathogens. The five mutations that directly contribute to sulfonamide resistance are boxed in red. Amino acids that are 100% conserved, highly conserved and weakly conserved are highlighted in dark blue, light blue, and gray, respectively.

A survey of other organisms was conducted to determine which of these mutations is conserved across species (Table 2). Mutations equivalent to F17L were found in *Neisseria meningitidis* and *Escherichia coli*, and mutations equivalent to T51M were found in *Plasmodium* species, *Pneumocystis carinii, Mycobacterium leprae*, and *Streptococcus pneumoniae* (Dallas et al., 1992; Fermer et al., 1995; Lane et al., 1997; Maskell et al., 1997; Wang et al., 1997b; Elena et al., 1998; Kazanjian et al., 1998; Mei et al., 1998; Kai et al., 1999; Williams et al., 2000; Pornthanakasem et al., 2016). A mutation homologous to E208K was also found in *Plasmodium* species but not in conjunction with any of the primary mutations (Pornthanakasem et al., 2016). We did not identify mutations equivalent to S18L or KE257_dup in other species. Alignment of DHPS sequences from *S. aureus* strains NCTC 8325 and Rosenbach 25923, and nine other microbial pathogens reveals that the primary mutations occur at highly conserved regions of the sequence while the secondary mutations occur in less conserved regions (Figure 1).

**Table 2:** DHPS mutations associated with sulfonamide resistance in *S. aureus*, and their homologs in *E. coli* (Dallas et al., 1992), *N. meningitidis* (Fermer et al., 1995), *B. anthracis* (Yun et al., 2012), *S. pneumoniae* (Maskell et al., 1997), *P. falciparum* (Wang et al., 1997a), *P. vivax* (Pornthanakasem et al., 2016), *M. leprae* (Kai et al., 1999), and *P. carinii* (Mei et al., 1998).

**Classification**	**Mutation**	**Location**	**Similar Mutations in Other Microbial Species**
Primary	F17L	Loop 1	*E. coli, N. meningitidis, B. anthracis*
	S18L	Loop 1	None
	T51M	Loop 2	S. pneumoniae^*^, P. falciparum^*^, P. vivax^*^, M. leprae^*^, P. carinii
Secondary	E208K	α-helix Loop 7	*P. falciparum*^†^, *P. vivax*^†^
	KE257_Dup	α-helix 8	None

*,† *Indicate that mutations align with T51M and E208K, respectively, but are not strictly conserved (Figure 1)*.

### Thermal stabilities of sulfonamide resistant SaDHPS mutants

To explore how the resistance mutations identified in this study affect the stability of *Sa*DHPS, the “wild type” enzyme from sulfonamide-susceptible strain Rosenbach 25923 and eight defined mutant enzymes were generated, expressed in *E. coli* and purified. Five of the mutant enzymes contained the single mutations F17L, S18L, T51M, E208K, and KE257_dup, and three contained the clinically observed double mutations F17L/E208K, T51M/E208K, and F17L/KE257_dup. The DHPS from sulfonamide susceptible *S. aureus* strain NCTC 8325 that harbors the 11 background variations not associated with sulfonamide resistance was also expressed and purified as a further control.

Thermal shift assays were employed to measure the denaturation temperatures (T_M_) of the purified proteins and to assess whether the mutations affect their stabilities (Table 3). These experiments were performed using Sypro-Orange that fluoresces when exposed to the hydrophobic interior of unfolded proteins upon denaturation. The *Sa*DHPS primary mutations did not significantly alter the T_M_, falling within 1°C of wild type 25923. In contrast, the secondary mutations E208K and KE257_dup both result in a 3–4°C drop in T_M_, revealing that these secondary mutations destabilize the protein structure. Addition of the F17L or T51M mutations to E208K maintains the 3–4°C drop in T_M_ whereas addition of the F17L mutation to KE257_dup restores the stability of the protein. These results are consistent with the *Sa*DHPS crystal structure (Hampele et al., 1997). F17, S18, and T51 are in the two flexible loops 1 and 2 that are disordered in the absence of substrates and unlikely to contribute to the stability of the protein fold. In contrast, E208 is part of a salt bridge array involving R176, R204, and K207 that appears to stabilize this region of the protein. The structural basis for the reduction in stability produced by the KE257_dup mutation is not so obvious and it may simply result from its location at the dimer interface. However, the compensation in stability provided by F17L suggests that it may involve the dynamic allosteric communication between the interface and the active site that we previously described (Hammoudeh et al., 2014). The DHPS from NCTC 8325 is ~4°C more stable than wild type DHPS from Rosenbach 25923, indicating that the 11 background mutations generate an inherently more stable enzyme.

**Table 3:** Changes in thermal stabilization of DHPS imparted by the observed sulfonamide resistant variations.

	**DHPS Variant**	**T_M_ (°C)**	**Δ T_M_ (°C)**
Rosenbach 25923		38.63 ± 0.09	
NCTC 8325		42.48 ± 0.01	
Primary mutation	F17L	39.14 ± 0.0	0.51
	S18L	39.52 ± 0.003	0.89
	T51M	38.7 ± 0.09	0.07
Secondary mutation	E208K	34.89 ± 0.04	−3.74
	KE257_dup	34.26 ± 0.1	−4.37
Double mutation	F17L E208K	34.77 ± 0.05	−3.86
	F17L KE257_dup	40.34 ± 0.14	1.71
	T51M E208K	35.67 ± 0.05	−2.96

*Changes in melting temperature (ΔT_M_) are relative to the Rosenbach 25923 wild type DHPS enzyme*.

### Catalytic properties of sulfonamide resistant SaDHPS mutants

We then analyzed the kinetic properties of the purified proteins (Table 4). The K_M_ values for DHPP, *p*ABA and SMX were measured using a colorimetric assay that monitors the release of pyrophosphate. The Ki values of SMX were derived from a radiometric assay that monitors the incorporation of ^14^C-labeled *p*ABA into the 7,8-dihydropteroate product. The Kcat values for *p*ABA and SMX were also derived from the colorimetric assay. The primary mutations F17L, S18L and T51M impart a slight increase in the K_M_ for DHPP, but significantly larger increases for *p*ABA. In contrast, the effects are reversed for the secondary mutations where the increases in the DHPP K_M_ values are more pronounced than those for *p*ABA. When the primary and secondary mutations are combined, they consistently lower the *p*ABA K_M_ values toward that of the wild type protein and increase the DHPP K_M_ values to those seen in the secondary mutations alone. As anticipated, the K_M_ and Ki values for SMX showed that the drug efficiently binds and inhibits the wild type enzyme. F17L, both alone and in combination with the two secondary mutations, decreases the binding and inhibition of SMX, but this was not the case with T51M where the effects were less obvious. S18L also significantly increased the K_M_ for SMX but it was not possible to measure the Ki value for technical reasons. The same was true for the secondary mutations alone.

**Table 4:** Kinetic characterization of *S. aureus* DHPS variants.

	**saDHPS Variant**	**Native Substrate**		**SMX Antibiotic**		**Substrate Turnover**	
		**K_m_ DHPP (μM)**	**K_m_*p*ABA (μM)**	**K_m_ SMX (μM)**	**K_i_ SMX (μM)**	**K_cat_*p*ABA (s^−1^)**	**K_cat_ SMX (s^−1^)**
	Wild Type	10.0 (±1.4)	3.1 (±0.9)	5.9 (±0.2)	1.3 (±0.5)	4.5 (±0.4)	2.2 (±0.1)
Primary mutation	F17L	18.3 (±4.7)	40.2 (±6.1)	202 (±54)	94.1 (±23.7)	1.7 (±0.3)	1.0 (±0.1)
	S18L	10.2 (±1.8)	26.2 (±0.2)	140 (±40)	ND	3.4 (±0.2)	1.6 (±0.1)
	T51M	12.1 (±2.6)	29.8 (±13.2)	3.9 (±1.6)	10.0 (±0.68)	1.9 (±0.2)	0.8 (±0.1)
Secondary mutation	E208K	34.5 (±2.2)	15.7 (±4.5)	ND	ND	3.1 (±0.0)	1.5 (±0.1)
	KE257_Dup	21.1 (±1.6)	5.3 (±3.0)	ND	ND	1.8 (±0.0)	0.9 (±0.2)
Double mutants	F17L E208K	21.3 (±6.8)	13.1 (±2.6)	167.5 (±43.6)	362.1 (±33.3)	0.5 (±0.0)	0.3 (±0.1)
	T51M E208K	38.1 (±1.5)	9.2 (±3.5)	5.7 (±3.0)	29.0 (±2.7)	0.5 (±0.1)	0.3 (±0.0)
	F17L KE257_Dup	22.3 (±5.6)	14.3 (±3.3)	26.0 (±6.0)	158.9 (±31.7)	3.6 (±0.3)	2.1 (±0.2)

*DHPP, pABA and SMX K_M_ and K_cat_ values were measured using a colorimetric assay that monitors pyrophosphate release. A radiometric assay that monitors ^14^C-labeled pABA incorporation into 7,8-dihydropteroate was used to determine the SMX K_i_ values*.

The kinetic data confirmed that SMX is a *bona fide* substrate of DHPS, although the turnover rates with the natural substrate *p*ABA, as reflected in the Kcat values, were consistently lower for all the variants. The individual mutations, both primary and secondary, decreased the turnover rates for both ligands, which confirms that the catalytic efficiency is compromised by each mutation. Somewhat surprisingly, apart from F17L/KE257_dup, there was an additive decrease in turnover rates for both *p*ABA and SMX when the primary and secondary mutations are combined. Thus, the “rescue” of the *p*ABA K_M_ by the secondary mutations does not translate into a similar rescue of Kcat.

### Resistant mutations and antibacterial MIC values

To determine the individual effects of the identified resistance mutations in *S. aureus*, we measured the MIC values of isogenic strains that only contained the mutant DHPS enzymes (Supplementary Table 1). It was first necessary to perform allelic replacements of the wild type *folP* gene with the mutant genes to generate the required strains in a USA300 AH1263 background, and this was successful for seven of the eight mutants that were biochemically analyzed. We failed to generate an isogenic strain containing the T51M/E208K double mutation after several attempts, and we therefore used the COL *S. aureus* strain that naturally harbors these mutations for select experiments. We and others have found that metabolic intermediates and nutrients in standard testing media can mask the action of antibiotics, including sulfonamides, and that minimal inhibitory concentration (MIC) determinations are more easily and accurately performed in minimal media (Zlitni et al., 2013; Zhao et al., 2016). We therefore measured the MICs of the nine *S. aureus* strains in minimal SSM9PR media lacking *p*ABA and several products of folate biosynthesis (Reed et al., 2015). We used chloramphenicol (CAM) as the control antibiotic that does not act through the folate pathway, and it has an MIC of 3.1–4.2 μg/mL for all strains tested. The results are summarized in Table 5. The most notable increase in resistance by a primary mutation occurred in the T51M mutant with dapsone, which is a structurally distinct member of the sulfonamide class. It is therefore significant that a homologous loop 2 mutation is also observed in *M. leprae* and two malarial strains (Table 2), for which dapsone is a common treatment option (Matsuoka, 2010).

**Table 5:** μg/ml MIC_80_ values for DHPS variants.

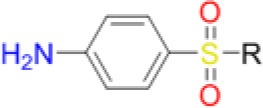		**Primary mutation**				**Secondary mutation**		**Double mutation**		
	**R**	**WT**	**F17L**	**S18L**	**T51M**	**E208K**	**KE257_dup**	**F17L E208K**	**F17L KE257_dup**	T51M E208K^*^
Sulfacetamide	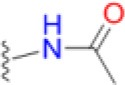	25	41.7	20.8	12.5	50	41.7	200	133.3	200 – >200
Sulfathiazole	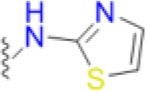	2.6	12.5	4.2	3.1	8.3	8.3	41.7	41.7	83.3
Sulfamethoxazole	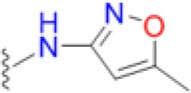	4.2	16.7	10.4	6.3	8.3	8.3	50	41.7	66.7
Sulfisoxazole	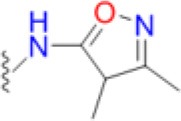	2.1	6.3	5.2	2.1	6.3	6.3	25	25	58
Sulfapyridine	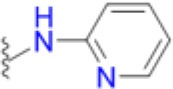	33.3	100	50	66.7	50	50	200	200 – >200	>200
Sulfadiazine	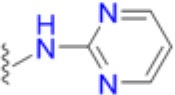	10.4	25	25	8.3	20.8	20.8	83.3	83.3	200
Dapsone	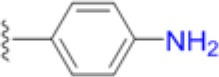	16.7	20.8	6.3	50	33.3	29.2	50	66.7	200 – >200
Sulfamethoxypyridazine	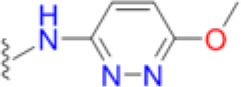	7.3	25.0	25.0	7.3	12.5	16.7	83.3	83.3	133.3
Sulfadimethoxine	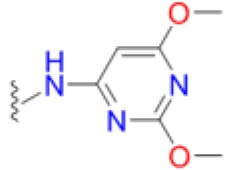	2.6	12.5	4.2	3.6	8.3	8.3	33.3	25	66.7
Sulfadoxine	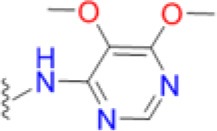	25	33.3	41.7	12.5	50	41.7	200	83.3	200 – >200
Chloramphenicol		4.2	4.2	3.1	3.1	3.1	3.1	3.1	3.1	3.1
Trimethoprim		1.6	0.065	0.024	0.16	1.6	1.0	0.16	0.20	2.1
$\frac{\text{Sulfamethoxazole}}{\text{Trimethoprim}}$^§^		$\frac{0.39}{0.021}$	$\frac{0.78}{0.041}$	$\frac{0.33}{0.017}$	$\frac{0.39}{0.021}$	$\frac{0.52}{0.027}$	$\frac{0.65}{0.034}$	$\frac{1.6}{0.082}$	$\frac{1.6}{0.082}$	$\frac{3.1}{0.16}$

MIC_80_ values reported are an average of three experiments. Apart from T51M/E208K, all mutants were cloned via allelic replacement into an isogenic USA300 AH1263 panel.

* *T51M/E208K values were derived from the COL S. aureus strain (Supplementary Table 1)*.

§ *The SMX/TMP combination MICs at the bottom of the Table represent co-trimoxazole at a ratio of 19:1*.

Individually, the primary mutations increased the MIC for most of the 10 sulfonamides tested in this study, but not markedly. F17L had the greatest impact, but only 4- to 5-fold for three of the sulfonamides. S18L and T51M had minimal effect, and the same was true for the two secondary mutations, apart from T51M with dapsone. In contrast, the combination of primary and secondary mutations dramatically increased the MIC values for all 10 sulfonamides. The largest effect was observed with T51M/E208K (Table 5), but this may be related to strain variability because this double mutation was evaluated in the COL *S. aureus* strain rather than as an allelic replacement in USA300 AH1263.

**Figure 2:**
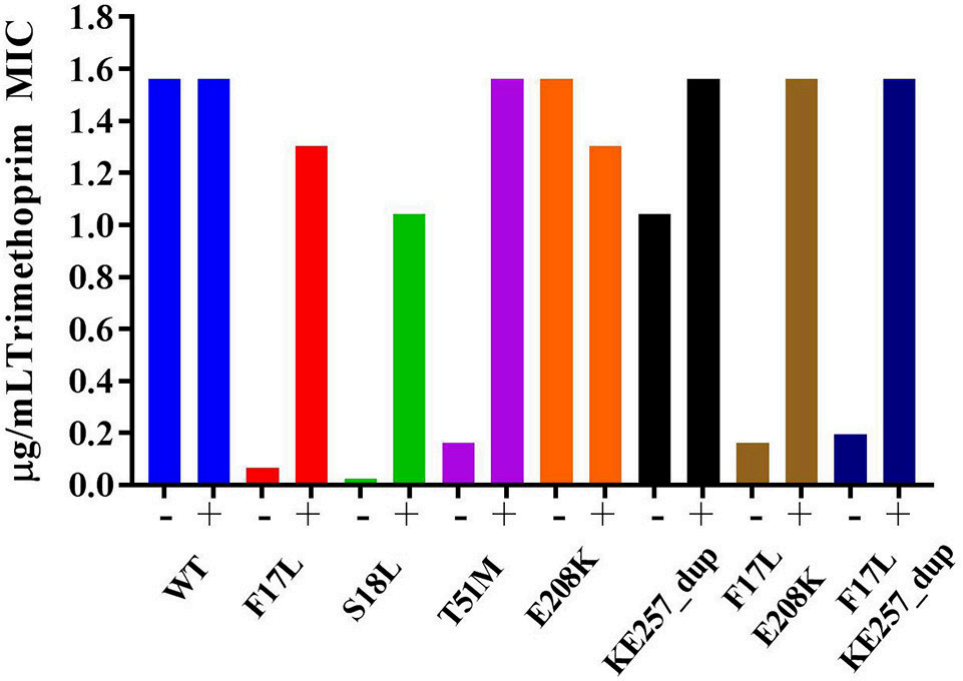
Trimethoprim susceptibility among mutants and influence of supplementation with 5 μg/mL *p*ABA. The presence and absence of *p*ABA in the testing media is indicated by + or – symbol, respectively. Data are representative of three independent experiments.

TMP targets DHFR within the folate pathway downstream of DHPS, and changes in the TMP MIC may indicate fitness costs associated with DHPS mutations. The primary resistance mutations significantly decreased TMP MIC, but the secondary mutations alone had negligible effect. When combined, the secondary mutations decreased the effect of the F17L mutant. Addition of *p*ABA to the testing media universally restored the TMP MICs closer to wild type for all the mutants. This could indicate that defects in catalysis caused by primary mutations in DHPS cause the downstream enzyme DHFR to become more crucial for overall pathway function, resulting in heightened susceptibility to DHFR inhibition as indicated by the lowered TMP MICs (Figure 2). The supplementation of *p*ABA may boost DHPS catalytic output, thus returning the TMP MICs to wild type levels. Appreciable increases in the co-trimoxazole SMX/TMP combination MICs were only observed with the double mutants, likely because the heightened TMP susceptibility of primary mutants drives the combination MIC closer to wild type levels despite having resistance to SMX (Table 5).

### Fitness studies of the folP mutants

**Figure 3:**
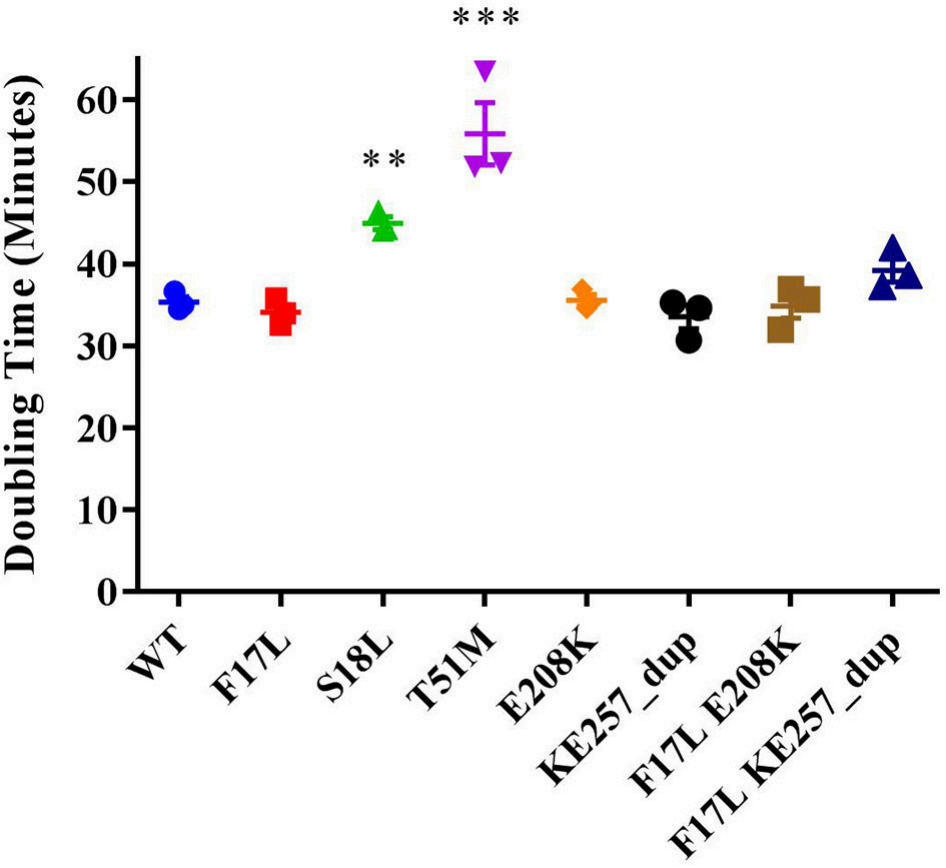
Doubling times for the isogenic USA300 *fol*P variant strains. **indicates *p* < 0.05, *** indicates *p* < 0.001 via ANOVA (Dunnett's multiple comparisons).

The kinetic data showed that the primary resistance mutations raise the K_M_ of *p*ABA and the secondary mutations lower the *p*ABA K_M_ back toward the value observed in the wild type enzyme. Furthermore, the TMP MIC testing revealed fitness consequences imposed by primary mutations at later stages of the folate biosynthesis pathway that could be restored by secondary mutation. Thus, the secondary mutations appear to restore the fitness of DHPS that is impaired by the primary mutations. To test whether these apparent changes in fitness of the isolated enzyme affect bacterial cell growth, the doubling times for the eight isogenic strains were measured (Figure 3). The strain harboring T51M had the slowest growth rate, but F17L did not have an altered growth rate. This was surprising because F17L had the highest consequence to *p*ABA binding (Table 4). However, the neighboring mutation S18L also had a significantly increased doubling time relative to the wild type enzymes. The remaining four mutations had no significant effect on doubling time.

**Figure 4:**
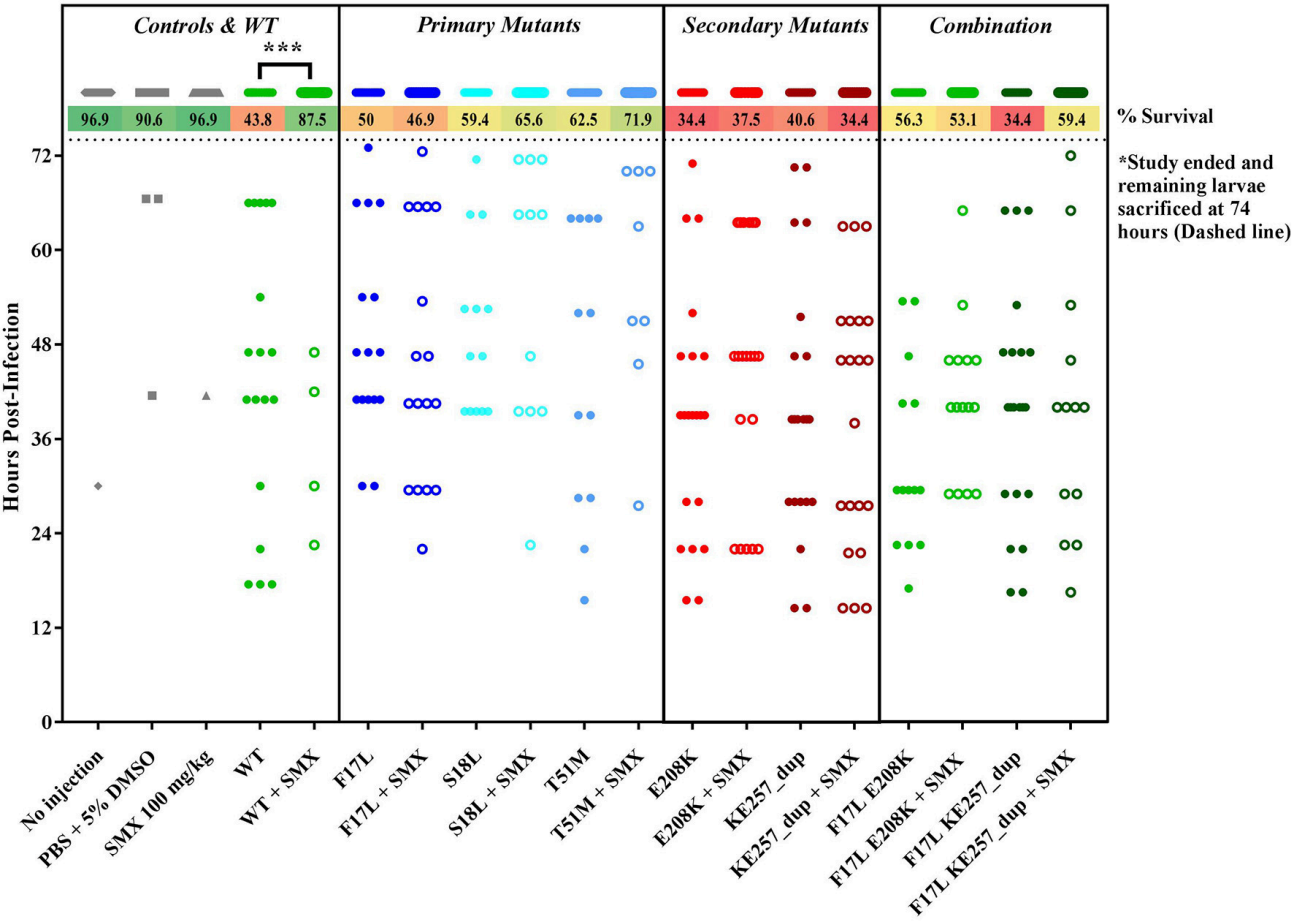
Wax moth larvae rescue study with isogenic USA300 *folP* variants. All groups contain 32 larvae and each data point indicates an observed death. Percentage survival at the end of the study is listed at the top. *** indicates *P* < 0.001 via Mantel Cox (Log-Rank) survival analysis.

To test whether the changes in fitness might become apparent *in vivo*, the isogenic *S. aureus* strains were subjected to a wax moth larvae infection study (Figure 4). In this model system, the larvae were infected with *S. aureus* strains from our isogenic panel of DHPS variants (Supplementary Table 1) and larval mortality rates were monitored. It was assumed that any significant decrease in the virulence of a particular strain would reflect the fitness cost associated with its introduced DHPS variant. Overall mortality rates at the end of the 72-h survival study indicated that the T51M mutant displayed the least virulence, consistent with the cell growth studies, but there were no significant changes in mortality-based virulence among any of the mutants according to Mantel Cox (Log-Rank) analysis. However, the wax moth larval model did respond as predicted in the presence of SMX. When administered 100 mg/kg SMX daily, the larvae could be rescued from the susceptible wild type strain, but every mutant strain was resistant to the antibiotic.

### Structural and biophysical analyses of the pABA/SMX binding pocket

**Figure 5:**
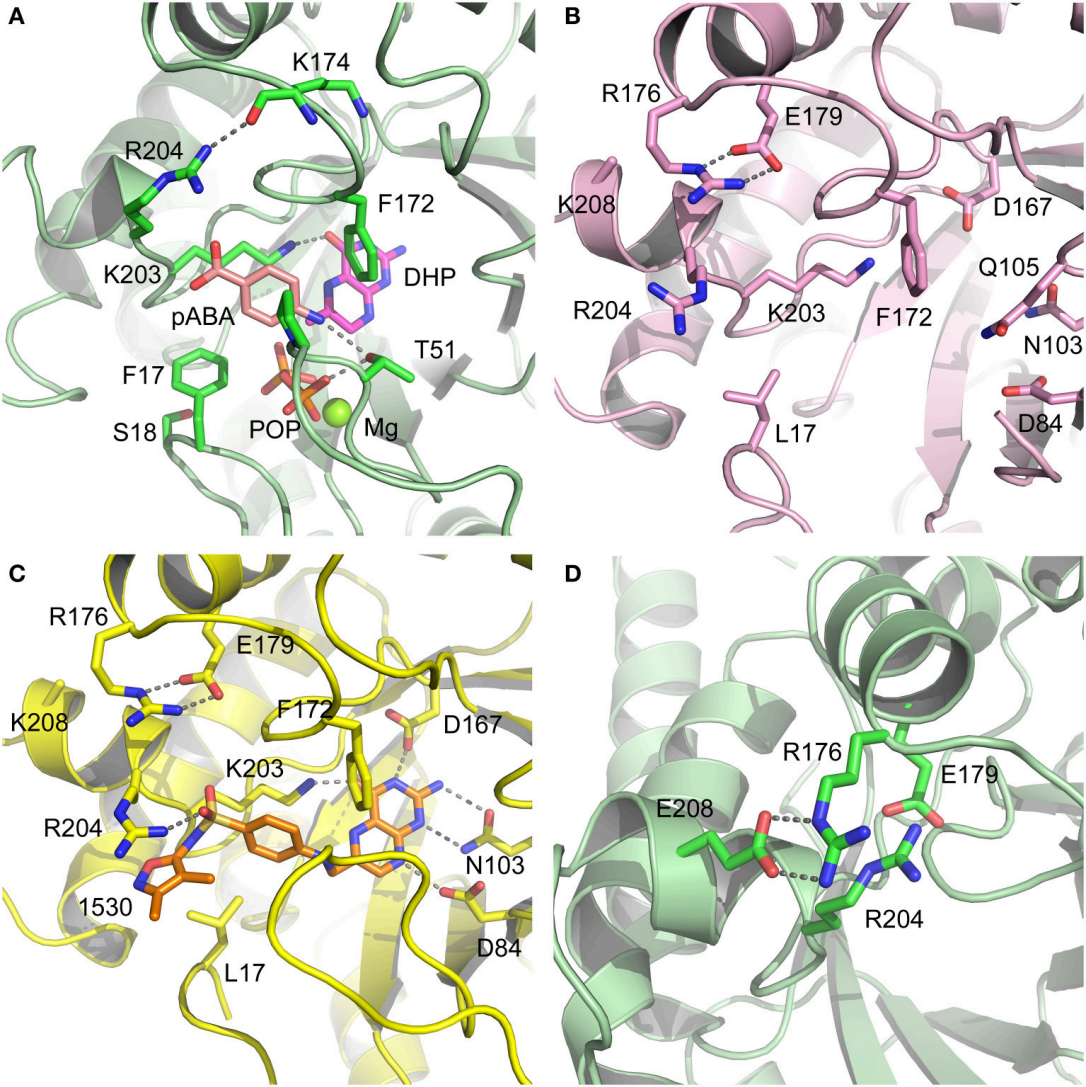
DHPS active site locale. **(A)** The modeled *S. aureus* DHPS transition state based on the published *Y. pestis* structure (3TYZ) highlighting the *p*ABA environment. The protein backbone is shown in pale green cartoon, the residues are in stick representation with green carbon, *p*ABA and DHP are in stick representation with salmon and magenta carbons, respectively, and pyrophosphate is orange. The essential Mg^2+^ ion is shown as a fluorescent green ball. **(B)** The mutated salt bridge arrangement in the crystal structure of *Sa*DHPS-F17L-E208K. The protein backbone is shown in purple cartoon, and the residues are in stick representation with purple carbons. **(C)** The mutated salt bridge arrangement in the crystal structure of the *Sa*DHPS-F17L-E208K-1530 compound complex. The protein backbone is shown in yellow cartoon, the residues are in stick representation with yellow carbons, and compound 1530 is in stick representation with orange carbons. **(D)** The wild type *Sa*DHPS salt bridge arrangement adjacent to the active site locale from the previously published crystal structure (1AD1). The coloring is the same as **(A)**. In all figures, the dashed gray lines indicate salt-bridges and hydrogen bonds.

Having revealed the importance of Phe17, Ser18, and Thr51 in sulfa drug resistance, we re-examined their structural roles in the context of the near-transition state structure. We first modeled and energy minimized the structures of the *Sa*DHPS near transition states in the presence of *p*ABA and SMX using our previously determined crystal structures of *Yp*DHPS (Yun et al., 2012). This proved to be very straightforward because the residues are highly conserved in the DHPS active site locale. Like all DHPS structures, loops 1 and 2 are disordered in the absence of substrates but become ordered in the near-transition state to create the *p*ABA-binding pocket and to structurally and chemically optimize the substrates for catalysis (Yun et al., 2012). This *Sa*DHPS active site locale is shown in Figure 5A, which highlights the roles of Phe17, Ser18, and Thr51. Phe17, together with Pro53, Phe172, and Lys203 create the *p*ABA-binding pocket, with the side chain rings of Phe17, Pro53, and Phe172 forming edge-to-face interactions with the phenyl ring of *p*ABA. The adjacent Ser18 does not interact with *p*ABA but appears to stabilize loop1 in this region. Meanwhile, the hydroxyl group of Thr51 forms hydrogen bonds with the amino group of *p*ABA and an oxygen of the pyrophosphate group that has been released from DHPP prior to the S_N_1 reaction that forms the product (Yun et al., 2012). Thr51 appears to help align the amino group for bond formation to the C11 carbon atom of the pterin substrate.

**Figure 6:**
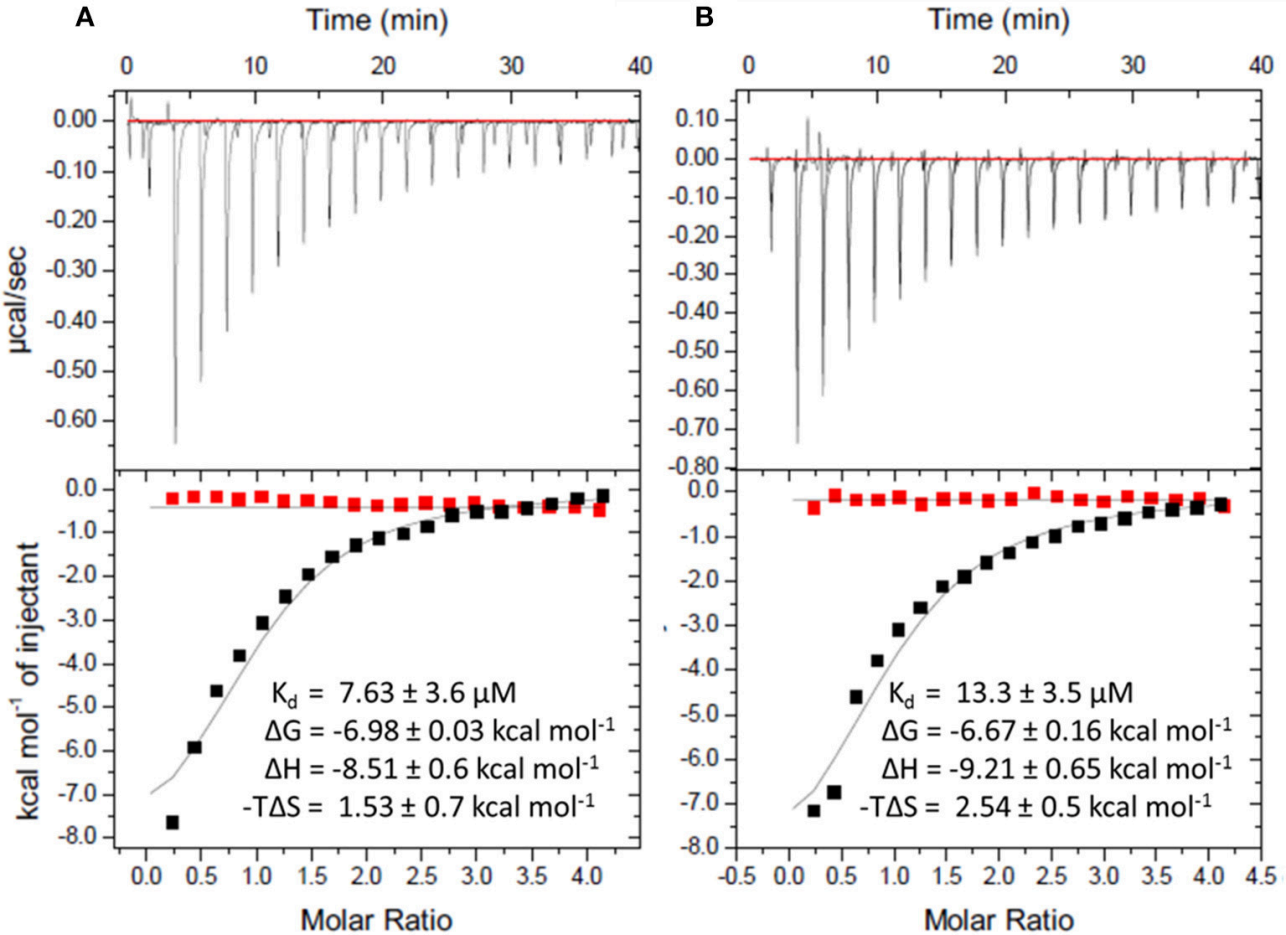
Isothermal titration calorimetric analysis of *p*ABA or SMX binding to DHPS in the presence and absence of sodium pyrophosphate. Trace of the calorimetric titration of 19 × 2 μl aliquots of 500 μM *p*ABA **(A)** or SMX **(B)** into 25 μM DHPS (top) and integrated binding isotherms (bottom). Red squares represent the heat of binding in the absence of sodium pyrophosphate. Black squares represent heat of binding in the presence of 10 mM sodium pyrophosphate. The solid black lines represent the best fit to a one site model. The derived thermodynamic parameters are shown as insets in the lower panel.

To gain more insights into the formation of the transition state ordered loop structure and the binding of *p*ABA and sulfonamides, we used isothermal titration calorimetry (ITC). ITC revealed that, while *p*ABA and pyrophosphate are both absolutely required to generate the *p*ABA-binding pocket, the pterin moiety of DHPP is not necessary (Figure 6). This is consistent with the ordered loop structure that makes multiple conserved interactions with the enclosed *p*ABA and pyrophosphate while the pterin moiety is independently accommodated in an adjacent preformed pocket (Figure 5A). The binding thermodynamics of SMX are almost identical to those of *p*ABA (Figure 6), which is consistent with our published structures that show that both occupy the binding pocket created by loops 1 and 2 in almost identical fashion (Yun et al., 2012). In the presence of pyrophosphate, *p*ABA and SMX have negative free energy (−6.9 and −6.8 kcal/mol respectively) reflecting a spontaneous binding interaction. The negative enthalpy measured in these studies is indicative of binding interactions driven by hydrogen bonding or π-stacking, again consistent with our structures. Finally, the significant entropic penalty associated with the binding of *p*ABA and SMX is consistent with the observed ordering of loops 1 and 2.

### Structural analyses of SaDHPS F17L/E208K

We crystallized *Sa*DHPS F17L/E208K and determined its structure in the absence of ligands (apo) and with our previously published (Zhao et al., 2016) inhibitory pterin-sulfisoxazole conjugate compound 1530 (Figures 5B,C, respectively). In the published *Sa*DHPS wild type structures, Phe17 within loop1 is either distant from the active site locale or missing (Hampele et al., 1997). However, in our two F17L/E208K structures, F17L and loop1 adopt a conformation that is similar but not identical to the known conformation of loop1 in the near transition state (compare the position of Phe17 in Figure 5A with the positions of Leu17 in Figures 5B,C). We have previously shown that compound 1530 binds to the wild type DHPS active site locale in a similar fashion to the pterin substrate and SMX in the near transition state (Yun et al., 2012; Zhao et al., 2016). In the F17L/E208K mutant structure complexed with compound 1530, Leu17 is directly adjacent to, and may therefore sterically impact, the oxazole ring of sulfisoxazole (Figure 5C).

The structural consequences of the E208K mutation are apparent from our two structures. In the wild type *Sa*DHPS structures, Glu208 forms a salt bridge with Arg176 and the adjacent Glu179 forms a salt bridge with Arg204 (Figure 5D). Arg176 and Arg204 also interact via a π-stacking interaction to stabilize this 4-residue cluster. When the E208K mutation is introduced, Arg176 relocates to form a salt bridge with Glu179 and Arg204 is displaced (Figures 5B,C). The structures suggest three ways in which the E208K mutation can contribute to resistance. First, the relocated Arg204 is adjacent to the oxazole ring in the 1530 complex (Figure 5C) and may sterically interfere with the transition state binding of sulfa drugs that have similar moieties (Table 5). The relocated Arg204 does not impact the phenyl ring of 1530 and should therefore have minimal impact on the binding of *p*ABA that occupies the same location. Second, the relocated Arg204 may form a stabilizing salt bridge with the carboxyl group of *p*ABA and thereby compensate for the negative impact on *p*ABA binding of the F17L and T51M mutations. The equivalent of this interaction with the negatively charged sulfone of sulfisoxazole is visible in Figure 5C. Finally, disruption of the Arg176/Glu179/Arg204/Glu208 salt bridge substructure may introduce some local flexibility to facilitate the distortions of the *p*ABA-binding site caused by the F17L, S18L, and T51M mutations. This is consistent with the thermal shift assay data for E208K (Table 3).

### Modeling studies of F17L, S18L, and T51M in the transition state

**Figure 7:**
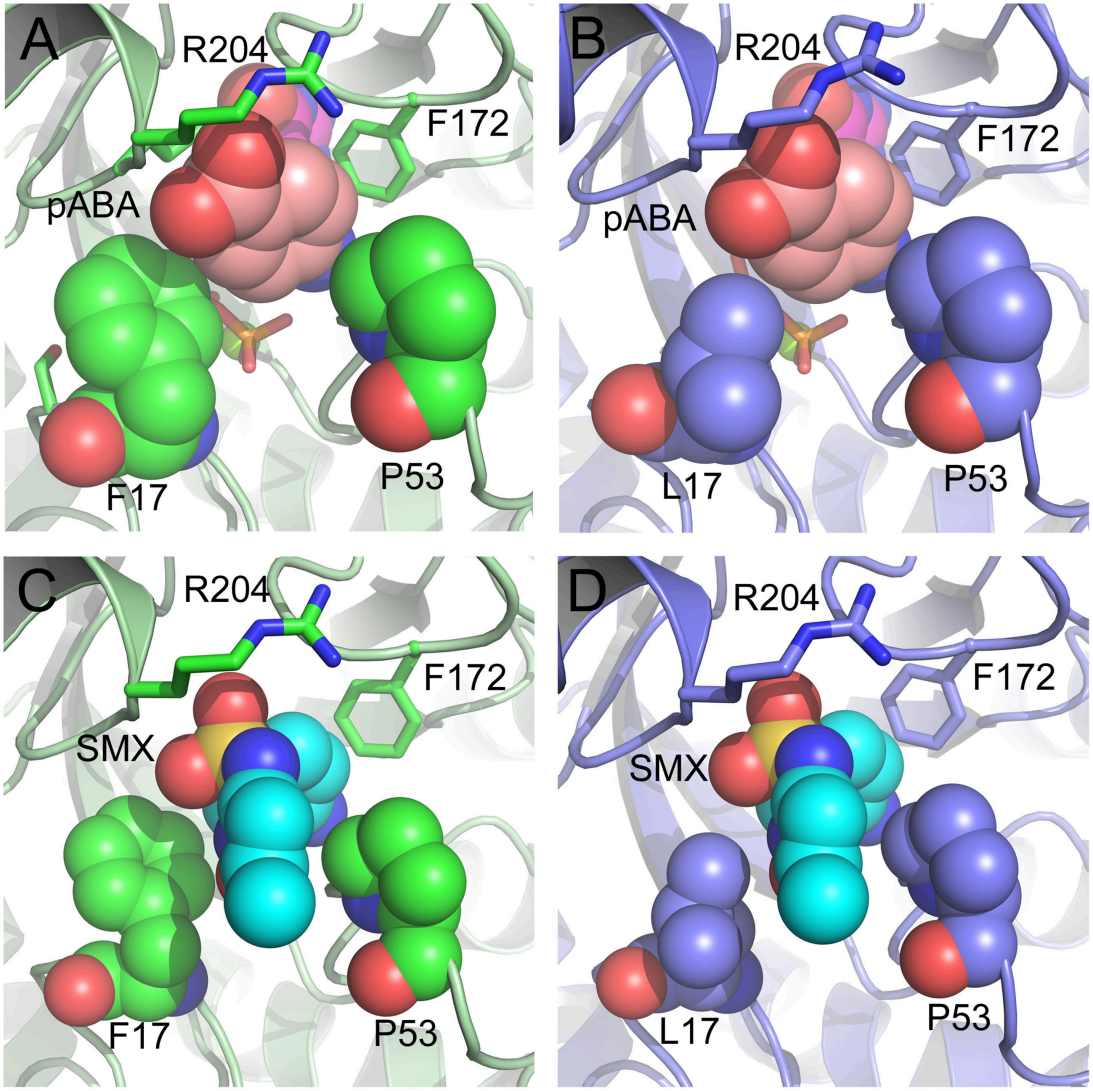
Close up view of the wild type and F17L mutant of *Sa*DHPS in complex with *p*ABA and sulfamethoxazole (SMX). **(A)** Wild type with *p*ABA. **(B)** F17L with *p*ABA. **(C)** Wild type with SMX. **(D)** F17L with SMX. Color scheme – wild type *Sa*DHPS, green carbons and ribbon; F17L *Sa*DHPS, violet carbons and ribbon; *p*ABA, salmon carbons; SMX, cyan carbons. The complexes were modeled using the *Yp*DHPS transition state complexes (PDB ID: 3TYZ and 3TZF).

We failed to obtain crystal structures of F17L, S18L, and T51M and we therefore turned to modeling and energy minimization to gain further insights into their roles in resistance. We introduced the three mutations independently into the two modeled *Sa*DHPS transition state structures containing *p*ABA or SMX, and performed energy minimization. The side chain of Leu17 is predicted to adopt the same rotamer in the *p*ABA and SMX complexes that minimally impacts the transition state structure. However, while this rotamer maintains a favorable and close interaction with *p*ABA, it sterically impacts the methylisoxazole ring of SMX. In the case of T51M, the mutation appears to have an indirect affect by impacting the location of Pro53 in loop2. As described above, Pro53 loosely forms part of the *p*ABA binding pocket, but it forms a tight van der Waals interaction with the methylisoxazole ring of SMX. The modeling suggests that any movement of Pro53 toward the methylisoxazole ring caused by T51M would selectively disfavor the binding of SMX compared to *p*ABA. Modeling with S18L did not yield a clear answer, but we suggest that it also acts indirectly to impact the position of the adjacent Phe17. The conclusions from these modeling studies are summarized in Figure 7.

## Discussion

The output of novel antibiotic classes has been greatly reduced in recent years, and there is a crucial need for new orally bioavailable antimicrobials effective against pathogenic Staphylococci such as MRSA and vancomycin-resistant *S. aureus* (VRSA). The folate biosynthesis pathway remains a viable target for inhibitor development due to the essentiality of this pathway in microbes. Indeed, sulfonamides that target the DHPS enzyme in the folate pathway remain a useful treatment option for common infection types such as UTIs, skin and soft tissue infections, and osteomyelitis (Liu et al., 2011). Opportunities for new therapies that target the folate pathway are afforded by continuing advances in our understanding of the catalytic mechanisms of DHPS and other component enzymes such as 6-hydroxymethyl-7,8-dihydropterin pyrophosphokinase (Shi et al., 2012; Dennis et al., 2016), and powerful new technologies to probe and exploit these mechanisms. In this study, we have analyzed the sequences of DHPS from sulfonamide resistant clinical isolates of *S. aureus* to identify the biochemical and structural basis of resistance. This information will be invaluable for developing new therapeutics that target DHPS and minimize the potential for resistance.

We have demonstrated using bioinformatics, biochemistry and microbiological analyses that five mutations in the enzyme DHPS directly contribute to sulfonamide resistance. We designated three of these mutations, Phe17, Ser18, and Thr51, as “primary” because one of these mutations is consistently present in resistant strains, and two mutations, E208K and KE257_dup, as “secondary” because they only appear in the presence of the primary mutations. Our crystallographic and modeling analyses reveal how these mutations achieve this resistance at the structural level. The three amino acids altered by primary mutations are highly conserved and have fundamental roles in creating the transition state structure in which two otherwise flexible loops become ordered by the pyrophosphate of DHPP and create a pocket that is bound and stabilized by *p*ABA (Yun et al., 2012). ITC data reported here confirm this mechanism. Sulfonamides are exquisite mimics of *p*ABA that can also engage this pocket, and we demonstrate by measuring the K_M_ values that the primary mutations have a greater negative impact on sulfonamide binding compared to *p*ABA. Previous studies have also reported this phenomenon (Fermer and Swedberg, 1997; Vedantam and Nichols, 1998; Gibreel and Sköold, 1999; Haasum et al., 2001; Levy et al., 2008). The secondary mutations partially restore *p*ABA binding to the wild type levels but do not restore the catalytic efficiency that is significantly reduced by the primary mutations. This is consistent with a recent study on clinical mutations in *Plasmodium* species that showed similar additive increases in sulfonamide K_i_ and whole-cell resistance (Pornthanakasem et al., 2016).

Thus, the mechanism of resistance is based on increasing the K_M_ of the sulfonamides compared to *p*ABA, and the organism can apparently survive with a less efficient DHPS enzyme under drug selective pressure. Our structural studies reveal that the discrimination between the binding of sulfonamides compared to *p*ABA is via the electron deficient outer ring, exemplified by methylisoxazole in SMX, that is required to generate the negative charge on the sulfone that mimics the carboxyl group of *p*ABA. As shown in Figures 5, 7, all three primary mutations appear to impact this ring and therefore the binding of the entire drug. The same is also true for the E208K secondary mutation that leads to a reorganization of a salt bridge structure adjacent to the active site locale that also appears to disfavor the binding of sulfonamides via this outer ring moiety. The way in which KE257_dup functions at the dimer interface is less apparent. We suggest that it operates allosterically based on our previous studies that identified inhibitory compounds that bind at the dimer interface, rigidify the dimer and significantly slow down the release of product (Hammoudeh et al., 2014). Overall, the data are most consistent for the F17L/E208K double mutant. The kinetics, sulfonamide susceptibility, crystal structures and modeling all present a consistent picture of how these two mutations work in concert to efficiently produce high levels of resistance. This double mutation results in a high level of resistance and is frequently observed in sulfonamide resistant strains of *S. aureus*.

Avoiding resistance is a key component of any successful drug discovery regime, as has been shown with other notoriously mutable targets such as the essential NS3/4A protease component of the hepatitis C virus (HCV). Structural studies with this protease indicated that the most marked increases in resistance are seen among inhibitors that extend beyond the “substrate envelope,” or consensus volume assumed by native substrate binding (Romano et al., 2010). Our studies reveal the same basic mechanism of resistance in DHPS but also show the importance of secondary mutations that can partially offset the negative impact of primary resistance mutations on enzyme function. Developing inhibitors that only occupy the volume assumed by native substrates will continue to be a key strategy in our drug discovery efforts on DHPS and other key enzymes in the folate pathway (Yun et al., 2014). An important conclusion from our studies is that continued development of the sulfonamide scaffold focused on the ring extending beyond the native substrate binding pocket is fundamentally flawed. Historically, structure activity relationship efforts to improve the efficacy of sulfonamides have focused on the outer ring which also produces more favorable potency and ADME properties. Although sulfanilamide and sulfacetamide lack this ring moiety, they are significantly less potent, and all potent sulfonamides are therefore inherently vulnerable to this mechanism of resistance (Greenwood, 2010).

Our studies demonstrate that the biochemical data derived from the resistance mutations do not necessarily translate into cellular MIC or fitness measurements. The flow of precursors from other metabolic pathways and uptake of exogenous metabolites contributes to resistance and fitness within the folate pathway. Our results also reflect that there is direct interplay between the enzymes within the folate pathway. Thus, we observed a modulation of TMP growth inhibition by both primary DHPS mutations and exogenously provided *p*ABA. This “*p*ABA rescue” effect has been observed with novel inhibitors generated by our group (Zhao et al., 2016), and with MAC173979, a *p*ABA biosynthesis inhibitor discovered by Zlitni et al. (2013). We also demonstrate that resistance to DHPS inhibitors increases sensitivity to TMP. This indirect consequence toward the susceptibility of the downstream enzyme DHFR is consistent with the mutual potentiation effects recently described between SMX and TMP (Minato et al., 2018). In these studies, TMP is shown to potentiate SMX by limiting production of the 7,8-dihydropteroate precursor DHPP, and SMX is shown to potentiate TMP by ultimately limiting 7,8-dihydrofolate production. The latter potentiation effect is similar to the results observed with the primary mutations in our study which hinder biochemical function.

Secondary mutations are not observed by themselves in our genetic survey of clinical isolates, perhaps due to its limited size. E208K when combined with F17L, clearly contributes to resistance and partially restores the binding of *p*ABA, and one might expect these benefits to be present without F17L. Our data show that this is not the case and that E208K fails to provide an advantage under the selective pressure of sulfonamide treatment.

Class modification is a highly successful strategy to develop improved antimicrobial agents that continue to engage clinically validated targets, but are engineered to avoid limiting resistance mechanisms (Silver, 2011). Many groups, including ours, are applying this strategy toward the folate biosynthesis pathway, within which DHPS remains a promising target (Bermingham and Derrick, 2002; Lombardo et al., 2016). Numerous pathogenic species acquire sulfonamide resistance through equivalent mutations to those we have characterized in this study. The findings from our work describes the structural and biochemical basis of sulfonamide resistance in *S. aureus*, which can now be used for the future development of next generation DHPS inhibitors suitable for the treatment of many multidrug-resistant pathogens.

## Materials and methods

### Genetic survey of foLP variations contributing to sulfonamide resistance

Comparative analyses of the DHPS amino acid sequences were performed against a set of 56 *S. aureus* reference strains and 80 non-redundant *S. aureus* DHPS sequences from GenBank using BLAST (Benson et al., 2005; Pruitt et al., 2005; Boratyn et al., 2012). Sequence variances in DHPS were recorded and compared. Based on these analyses, sequences were separated into the two wild type backgrounds, and 8 subgroups containing at least one of the 5 variations that contribute to sulfonamide resistance or a combination of them. Sulfonamide susceptibility data for each isolate, where available, were associated with the sequencing data. Amino acid sequence alignments were performed using Clustal Omega (Goujon et al., 2010; Sievers et al., 2011).

### SaDHPS expression and purification

The *folP* gene of *S. aureus* Rosenbach 25923 DHPS (EMBL accession number Z84573) and those of the F17L, S18L, T51M, E208K, KE257_dup, T51M/E208K, F17L/E208K, and F17L/KE257_dup mutants were synthesized into the pET16b vector by GenScript to produce the protein with a N-terminal 10xHis tag. These plasmids were used to transform competent *E. coli* BL21[DE3] cells. Resultant cell lines were used to inoculate 1L LB broth containing 50 μg/mL Ampicillin (added as a selective agent). These cultures were incubated at 37°C with shaking at 225 RPM until they reached an OD_600_ of 0.5. These cultures were then induced with 1 mM IPTG and incubated overnight at 18°C with shaking at 225 rpm. Cell pellets were collected with centrifugation at 3700 RCF and resuspended in a lysis buffer consisting of 50 mM Tris pH 8, 500 mM NaCl, 5 mM imidazole, lysozyme, and protease inhibitor cocktail (Roche 11-836-170-001). Cells were lysed by sonication and cell debris was cleared with centrifugation at 14,000 rpm. Crude lysate was further clarified by filtration through a 0.22 μm filter. DHPS was purified from crude lysate in two steps. Crude lysate was applied to a GE HisTrap HP 5 ml column at a rate of 0.2 ml/min. The column was then washed with 200 ml Buffer A consisting of 50 mM Tris, 500 mM NaCl, and 5 mM imidazole, pH 8. DHPS was eluted from the GE HisTrap HP 5 ml column using a gradient of 5–500 mM imidazole. Elution fractions were collected and those with an elevated UV spectrum at 280 were pooled. Pooled fractions were then applied to a HiPrep 16/60 Sephacryl S-200 HR column at a rate of 0.5 ml/min. The column was then washed with 2 column volumes elution buffer consisting of 50 mM HEPES, 150 mM NaCl, and 1 mM DTT at pH 7.6. Elution fractions were collected and examined via SDS-PAGE. Those fractions yielding a single band at approximately 32 kDa were pooled as a final product of purification. Samples were flash frozen using liquid nitrogen and stored for a maximum of 6 months at −80°C.

### DHPS kinetic characterization

All kinetic characterization experiments were carried out in 50 mM HEPES with 10 mM MgCl_2_ at pH 7.6. Two kinetic analyses were employed. The first measures the pyrophosphate that is released by the DHPS reaction. The pyrophosphate is converted to orthophosphate using yeast inorganic pyrophosphatase, and the PiColor Lock Gold assay (Innova Biosciences) was used to detect orthophosphate. The K_M_ values for the two substrates *p*ABA and DHPP were individually measured by maintaining one of the substrates at a concentration that was at least 20-fold in excess of the established K_d_.

The second kinetic analysis employed a radiometric assay that measures ^14^C-labeled *p*ABA incorporation into the 7,8-dihydropteroate product. The reaction was carried out for 30 min at 37°C in a total volume of 100 and 10 μl aliquots were removed at intervals of 5 min. Ice-cold 50% acetic acid was added to stop the reaction. Reaction products were loaded onto PEI TLC cellulose plates (Analtech 205016) followed by development in 100 mM NaPO_4_, pH 7. Phos-Screen exposure was followed by Typhoon imaging. Inhibition constants were determined by maintaining substrate levels at their K_d_. SMX was added at concentration ranges between 0 and 10 mM. The K_i_ values were determined using the one-site Fit K_i_ equation.

### Thermal stability studies

The stability of wild-type and variant DHPS was assessed using a thermal stability assay. Sypro-Orange was added to a 10 μM sample of DHPS in the same buffer conditions used for kinetic studies, resulting in a final concentration of 5X. The fluorescence of the solution was then measured over a range of temperatures (23–99°C). Resultant data were fit to the Boltzmann equation resulting in the melting temperature of the protein (T_M_).

### Isothermal titration studies

Binding of *p*ABA or SMX to DHPS in the presence or absence of 10 mM sodium pyrophosphate was performed on an iTC200 (MicroCal) at 25°C. Wild type DHPS was dialyzed into 2 L ITC buffer (50 mM HEPES pH 7.6, 5 mM MgCl_2_) overnight at 4°C. Standard ITC experiments were performed in 50 mM HEPES, 5 mM MgCl_2_, and 2.5% DMSO. The iTC200 Microcalorimeter was set to deliver 19 × 2 μl injections of 500 μM ligand at 150 s intervals into 200 μl of 25 μM protein solution. All experiments were completed in triplicate. Data were analyzed using MicroCal Origin 7.0 software using a one-site binding model with n values fixed at 1.

### Allelic replacement to generate isogenic USA300 AH1263 foLP mutants

The gene encoding *S. aureus folP* from wild-type strain NCTC 8325 was cloned into the PCR2.1TOPO vector. Site-directed mutagenesis was employed to generate eight variants of this gene with confirmed sulfonamide-resistance mutations present singly or in their observed clinical combination which included F17L, S18L, T51M, E208K, KE257_dup, F17L/E208K, F17L/KE257_dup, and T51M/E208K. These genes were sub-cloned into the shuttle vector pJB38, which has a temperature-sensitive *S. aureus* origin of replication. After transformation of *S. aureus* USA300 AH1263 with the pJB38 plasmids, growth at 43°C in the presence of chloramphenicol (CAM) allowed for plasmid integration, after which the bacteria were sub-cultured and allowed to grow at 30°C. Growth on anhydrous tetracycline allowed for the killing of cells that still contained the pJB38 plasmid. Absence of the plasmid was further confirmed by testing for sensitivity to CAM followed by sequencing of the *fol*P gene, which was PCR amplified from the genomic DNA of each mutant. All of the isogenic mutants were successfully generated with the exception of T51M/E208K.

### Minimum inhibitory concentration assay (MIC)

MIC testing was carried out in SSM9PR media (Reed et al., 2015). When necessary, *p*ABA was added to achieve a final concentration of 5 μg/ml. Antifolates were serially diluted in 100 μl/well 2-fold across 96 well plates. Colonies of each *S. aureus* wild type or mutant strain to be tested were resuspended in media to a mid-log OD_600_ between 0.3 and 0.6, after which they were further diluted to an OD_600_ of 0.001, which allowed for 100 ul of bacterial suspension containing 10^5^ CFU to be added to each well. After 16 h of incubation at 37°C, the concentration at which 80% growth inhibition had occurred was determined visually.

### Growth curve analysis

Each strain studied was streaked onto LB agar and grown overnight. The overnight cultures were further diluted 1:100 in LB and the OD_600_ was read every 30 min. Doubling times were calculated using the linear range of the growth curve of each mutant using the following equation (Reeve et al., 2016):

$$\begin{array}{l} Doubling\;time=\;\frac{\Delta Time\;x\;log2}{\text{log}\left(Final\;conc.\right)-\text{log}\left(initial\;conc.\right)} \end{array}$$

The growth curve experiment was performed with three biological replicates for each mutant. The calculated doubling times were compared using One-Way ANOVA Dunnett's Multiple Comparisons Test.

### Wax moth larvae infection study

*Galleria mellonella* larvae were purchased from Fisher Scientific (14-726-369) in their final instar stage. Larvae were stored at 13°C prior to experimentation and used within seven days of receipt. The larvae used in each experiment were obtained in a single batch and normalized for size. Each sample group contained 32 subjects. Each experiment had at least two control groups including a group that was maintained with no manipulation and a group that was injected with the vehicle solution 10 μl PBS/5% DMSO. For the rescue experiments, larvae were injected with SMX at 100 mg/kg daily in PBS/5% DMSO solution. To prepare the inoculum, *S. aureus* strains with chromosomal DHPS mutations of interest were streaked onto LB agar and incubated at 37°C overnight. A single colony was then used to inoculate 5 ml LB broth which was brought to mid-log at 37°C with shaking at 225 RPM. Cells were collected via centrifugation and suspended in PBS. Cell density was normalized to yield a 10 μl injection volume containing 5.9 × 10^5^ CFU, which has an LD_50_ at approximately 72 h for the wild type strain. During the course of the experiment, larvae were contained in petri dishes at 37°C. Over a period of 72 h, sample groups were checked for mortality, which was defined by lack of motility and alteration in color. Mortality data were analyzed using GraphPad Prism, with the Log-rank (Mantel-Cox) test used to determine statistically significant changes in mortality for each mutant compared to the wild type strain. The same statistical test was used to determine significant alterations in mortality due to SMX treatment for each strain tested.

### Crystallography

*Sa*DHPS-F17L/E208K was brought to a concentration of 20 mg/ml in crystallization buffer (50 mM HEPES pH 7.6, 150 mM NaCl, and 1 mM DTT). Initial crystallization conditions were determined using the JCSG I-IV screens wherein 200 nl of *Sa*DHPS were added to 200 nl of well solution in a sitting drop plate and incubated at 8°, 18°, and 23°C. Initial crystals were optimized to produce the final conditions of 0.2 M sodium nitrate, 20% PEG 3350, 75 mM NaCl, 25 mM HEPES, pH 7.6 at 18°C. Crystals were soaked overnight in compound 1530 at a 3:1 molar ratio to generate the complex structure. Twenty-five percent glycerol was added to the crystallization buffer as a cryoprotectant, and crystals were flash frozen in liquid nitrogen prior to data collection at 100°K. X-ray data were collected at Southeast Regional Collaborative Access Team (SER-CAT) 22-ID beamline at the Advanced Photon Source, Argonne National Laboratory. Diffraction images were indexed and integrated with XDS (Kabsch, 2010), and scaled and merged with aimless (CCP4) in space group P43. The structure was solved with PHASER (McCoy et al., 2007) using the published *Sa*DHPS coordinates (PDB 1AD4) as the search model. Reciprocal space refinement was carried out in PHENIX (Afonine et al., 2012) using twin law h,-k,-l, NCS restraints, and TLS definitions in the final cycles. Ligand restraints were created with eLBOW (Moriarty et al., 2009) and real space refinement was performed using COOT (Emsley and Cowtan, 2004). Both structures have been deposited in Protein Data Bank with accession codes 6CLU and 6CLV. Data collection and refinement statistics are reported in Supplementary Table 2.

### Modeling and computational studies

The *Sa*DHPS transition state complexes with *p*ABA and sulfamethoxazole were modeled based on the crystal structures of *Yersinia pestis* DHPS in the near transition state complex with *p*ABA (3TYZ) and sulfamethoxazole (3TZF) using the program COOT (Emsley and Cowtan, 2004) and energy minimized using the program MOE (Molecular Operating Environment [MOE], 2018). The Amber ff12 force field was used for the protein and OPLS-AA for the small molecules. The system was energy minimized to 0.1 kcal/mol/A^2^.

## Author contributions

RL, SW, and CR oversaw and directed the research. They also contributed to the writing of the manuscript. EG performed the kinetic experiments, isothermal calorimetry, genetic survey of folP genes in *S. aureus*, and crystallized as well as collected the X-ray data. She also contributed to writing the manuscript. MW participated in generating the isogenic USA300 mutant panel, performed the minimal inhibitory concentration testing, and fitness studies. She also contributed to writing the manuscript. YW and GK processed the X-ray crystallography data and refined the structures. SG performed the modeling studies and contributed to writing the manuscript. PJ participated in generating the isogenic USA300 mutant panel. ZZ performed the initial bioinformatics analysis. GP participated in the wax moth larvae fitness study and helped to analyze the data.

### Conflict of interest statement

The authors declare that the research was conducted in the absence of any commercial or financial relationships that could be construed as a potential conflict of interest.
