# Supplementary material for: The Structural and Functional Basis for Recurring Sulfa Drug Resistance Mutations in Staphylococcus aureus Dihydropteroate Synthase
Source: Front Microbiol. 2018 Jul 17;9:1369. doi: 10.3389/fmicb.2018.01369 (PMC6057106; doi:10.3389/fmicb.2018.01369)
Supplement: Supplementary file 1 [file Table_1.docx]

**The Structural and Functional Basis for Recurring Sulfa Drug Resistance Mutations in** ***Staphylococcus aureus* Dihydropteroate Synthase**

Elizabeth C. Griffith^1‡^, Miranda J. Wallace^1,4‡^, Yinan Wu^2,†^, Gyanendra Kumar^2^,

Stefan Gajewski^2,#^, Pamela Jackson^3^, Gregory A. Phelps^1,5^, Zhong Zheng^1^,

Charles O. Rock^3^, Richard E. Lee^1,*^ and Stephen W. White^2,4,*^

**Supplementary Table 1**

Supplementary Table 1. Strains used in this study. The USA300 AH1263 isogenic panel of DHPS mutants has the DHPS background amino acid sequence from NCTC 8325. Variations in the DHPS amino acid sequence observed in Rosenbach 25923, another sulfonamide susceptible strain of *S. aureus*, are noted in the Table.

| **Strain Type** | **Name** | **Primary Mutation** | **Secondary**  **Mutation** | **Background Variations Relative to NCTC 8325** |
| --- | --- | --- | --- | --- |
| WT | Rosenbach 25923* | - | - | I30V, N31T, I37M, V58I, S59T, L60V, M64L, M101I, I117V, I126V, L266F |
| USA300 AH1263 Isogenic Panel | WT | - | - | - |
|  | F17L | F17L | - | - |
|  | S18L | S18L |  |  |
|  | T51M | T51M | - | - |
|  | E208K | - | E208K | - |
|  | KE257_dup | - | KE257_dup | - |
|  | F17L E208K | F17L | E208K | - |
|  | F17L KE257_dup | F17L | KE257_dup | - |
| Clinical Isolate | COL | T51M | E208K | - |
