# Supplementary material for: The Structural and Functional Basis for Recurring Sulfa Drug Resistance Mutations in Staphylococcus aureus Dihydropteroate Synthase
Source: Front Microbiol. 2018 Jul 17;9:1369. doi: 10.3389/fmicb.2018.01369 (PMC6057106; doi:10.3389/fmicb.2018.01369)
Supplement: Supplementary file 2 [file Table_2.docx]

**The Structural and Functional Basis for Recurring Sulfa Drug Resistance Mutations in** ***Staphylococcus aureus* Dihydropteroate Synthase**

Elizabeth C. Griffith^1‡^, Miranda J. Wallace^1,4‡^, Yinan Wu^2,†^, Gyanendra Kumar^2^,

Stefan Gajewski^2,#^, Pamela Jackson^3^, Gregory A. Phelps^1,5^, Zhong Zheng^1^,

Charles O. Rock^3^, Richard E. Lee^1,*^ and Stephen W. White^2,4,*^

**Supplementary Table 2**

Supplementary Table 2. Crystallography data collection and refinement statistics.

|  | **F17L/E208K** | **F17L/E208K-Lee1530** |
| --- | --- | --- |
| **Data Collection** |  |  |
| Wavelength (Å) | 0.979430 | 0.979180 |
| Resolution range (Å) | 46.62 - 1.95 | 47.4 - 2.30 |
| Space group | P4_3_ | P4_3_ |
| Unit cell dimensions (Å) | 76.64, 76.64, 176.23 | 79.75, 79.75, 175.40 |
| Total reflections | 684630 | 201103 |
| Unique reflections | 73686 | 48528 |
| Multiplicity | 9.2 (7.0) | 4.2 (2.9) |
| Completeness (%) | 99.9 (98.4) | 99.7 (96.6) |
| Mean I/sigma(I) | 9.7 (1.0) | 10.8 (1.2) |
| R-merge | 13.3 (194.8) | 6.8 (66.6) |
| R-meas | 15.1 (228.3) | 8.9 (87.9) |
| R-pim | 6.9 (116.5) | 5.6 (56.6) |
| CC1/2 | 0.997 (0.191) | 0.997 (0.474) |
| **Refinement** |  |  |
| Twin law | h,-k,-l | h,-k,-l |
| Twin fraction | 0.32 | 0.64 |
| Reflections used | 73613 | 48470 |
| Reflections in Rfree | 2005 (143) | 2546 (136) |
| R-work | 0.1819 (0.3928) | 0.1929 (0.2839) |
| R-free | 0.2036 (0.4370) | 0.2086 (0.3238) |
| Protein atoms | 7055 | 7472 |
| Ligand atoms | n/a | 93 |
| Solvent atoms | 197 | 105 |
| RMS (bonds) | 0.001 | 0.003 |
| RMS (angles) | 0.371 | 0.612 |
| Ramachandran favored (%) | 99.34 | 99.39 |
| Ramachandran allowed (%) | 0.66 | 0.61 |
| Ramachandran outliers (%) | 0.0 | 0.0 |
| Rotamer outliers (%) | 0.0 | 0.0 |
| Clashscore | 1.36 | 7.22 |
| Average B-factors |  |  |
| protein | 32.23 | 51.41 |
| ligand | n/a | 48.12 |
| solvent | 32.33 | 46.53 |
